# Sex Differences Across Concussion Characteristics in US Service Academy Cadets: A CARE Consortium Study

**DOI:** 10.1007/s40279-024-02068-3

**Published:** 2024-07-12

**Authors:** Louise A. Kelly, J. B. Caccese, D. Jain, C. L. Master, L. Lempke, A. K. Memmini, T. A. Buckley, J. R. Clugston, A. Mozel, J. T. Eckner, A. Susmarski, E. Ermer, K. L. Cameron, S. Chrisman, P. Pasquina, S. P. Broglio, T. W. McAllister, M. McCrea, C. Esopenko, CARE Consortium Investigators

**Affiliations:** 1 Department of Exercise Science, California Lutheran University, #3400, 60 W. Olsen Road, Thousand Oaks, CA 91360 USA; 2 College of Medicine School of Health and Rehabilitation Sciences, The Ohio State University, Columbus, OH USA; 3 Department of Rehabilitation and Human Performance, Icahn School of Medicine at Mount Sinai, New York, NY USA; 4 University of Pennsylvania Perelman School of Medicine, Philadelphia, PA USA; 5 Michigan Concussion Center, University of Michigan, Ann Arbor, MI USA; 6 Department of Health, Exercise and Sports Sciences, University of New Mexico, Albuquerque, NM USA; 7 Department of Kinesiology and Applied Physiology, University of Delaware, Newark, DE USA; 8 Department of Community Health and Family Medicine, University of Florida, Gainesville, FL USA; 9 Center for Injury Research and Prevention, Children’s Hospital of Philadelphia, Philadelphia, PA USA; 10 Physical Medicine and Rehabilitation, Michigan Medicine, University of Michigan, Ann Arbor, MI USA; 11 Medical Associates Clinic, Loras College, Dubuque, IA USA; 12 Physical Medicine and Rehabilitation, Uniformed Services University of the Health Sciences, Bethesda, MD USA; 13 Orthopaedic and Sports Medicine, United States Military Academy, West Point, NY 10996 USA; 14 Division of Adolescence Medicine, Department of Pediatrics, Seattle Children’s Hospital, Seattle, WA 98105 USA; 15 Department of Psychiatry, Indiana University School of Medicine, Indianapolis, IN USA; 16 Department of Neurosurgery, Medical College of Wisconsin, Milwaukee, WI USA

## Abstract

**Objective:**

To describe sex differences in concussion characteristics in US Service Academy cadets.

**Design:**

Descriptive epidemiology study.

**Setting:**

Four US service academies.

**Participants:**

2209 cadets (*n* = 867 females, *n* = 1342 males).

**Independent Variable:**

Sex.

**Outcome Measures:**

Injury proportion ratios (IPR) compared the proportion of injuries by sex (females referent) for injury situation, certainty of diagnosis, prolonged recovery, recurrent injuries, mental status alterations, loss of consciousness (LOC), posttraumatic amnesia (PTA), retrograde amnesia (RGA), motor impairments, delayed symptom presentation, and immediate reporting.

**Main Results:**

Concussions from varsity/intercollegiate sports [IPR of 1.73, 95% confidence interval (CI) 1.43–2.10] and intramurals (IPR of 1.53, 95% CI 1.02–2.32) accounted for a larger proportion in males, whereas concussions outside of sport and military activities accounted for a smaller proportion among males (IPR of 0.70, 95% CI 0.58–0.85). The proportion of concussions with prolonged recovery was lower among males (IPR of 0.69, 95% CI 0.60–0.78), while concussions with altered mental status (IPR of 1.23, 95% CI 1.09–1.38), LOC (IPR of 1.67, 95% CI 1.17–2.37), PTA (IPR of 1.94, 95% CI 1.43–2.62), and RGA (IPR of 2.14, 95% CI 1.38–3.31) accounted for a larger proportion among males. A larger proportion of concussions that were immediately reported was observed in males (IPR of 1.15, 95% CI 1.00–2.31). Proportions of other characteristics (e.g., recurrent injuries) were not different between sexes.

**Conclusions:**

A higher proportion of concussions occurred outside of sport and military training for female cadets, who also displayed proportionally longer recovery times than males, despite males demonstrating a higher proportion of LOC, PTA, and RGA. Possible factors may include different mechanisms of injury outside of sport and military training, different biopsychosocial states associated with sex or injury context, and delayed injury reporting when outside of an observed environment, possibly secondary to perceived stigma about reporting injuries.

**Supplementary Information:**

The online version contains supplementary material available at 10.1007/s40279-024-02068-3.

## Key Points

**Table:** 

This study addresses sex differences in concussion characteristics among military cadets.
Female cadets sustain a higher proportion of concussions outside of sport and military training.
Female cadets take longer to report concussion symptoms.

## Introduction

Concussion and return to play research and guidelines have traditionally focused on males. In recent years there has been an increase in the number of studies examining sex differences in the prevalence of concussions and the differential effects on physical, psychological, and cognitive health outcomes in athletes. Such studies in high school and collegiate athletes demonstrate mixed findings on sex differences in concussion rates and outcomes. Indeed, some studies demonstrate a higher prevalence of concussion as well as protracted recovery patterns including, but not limited to, symptom resolution, return to play, or return to learn among female high school and collegiate athletes [1–3]. Further, a recent large Concussion Assessment, Research, and Education (CARE) Consortium study concluded sex differences may be specific to contact-sport level (i.e., contact versus limited contact sports). Other studies, however, have shown no sex differences in prevalence of concussion particularly when athletes have similar access to medical services and care providers after injury [4, 5]. Sex differences in postconcussion injury characteristics, including loss of consciousness (LOC) and post-traumatic (PTA) and retrograde amnesia (RGA), have also been shown with male athletes exhibiting greater proportions of concussions with LOC, PTA, and RGA [6, 7]. Other concussion characteristics, such as the injury situation and contact-sport level, have also been shown to affect concussion prevalence and postinjury characteristics. Specifically, concussions more commonly occur in competition compared with practice and athletes participating in contact sports demonstrate a higher proportion of concussions with postinjury characteristics than limited and noncontact athletes. [6, 8, 9]

Concussions are also a major concern among military cadets enrolled in the US service academies, due to their inherent risk of head impacts during sport and military-specific training drills [10, 11]. Prior investigations across the CARE Consortium suggest a higher proportion of military cadets who are National Collegiate Athletic Association (NCAA) athletes, are younger (i.e., first year), female, or have a history of concussion and are likely to sustain concussions; however, cadets who also identify as athletes are faster to recover from concussions than their nonathlete counterparts [10, 12–14]. Furthermore, prior research suggests female cadets take longer to complete return to play protocols and demonstrate different reporting behaviors, which can affect recovery trajectories compared to male cadets [15, 16]. To the best of our knowledge, there are no studies to date that have addressed potential sex differences in concussion characteristics among military cadets. Understanding sex differences in situational factors that increase risk of concussion and negative outcomes may help us interpret mixed findings, as well as aid in future postinjury intervention.

Identifying sex differences in concussion characteristics in military cadets is ever important, particularly given the increasing representation of females within the service academies and, more broadly, the higher proportion of female active-duty service members being combat deployed. Thus, the goal of this study is to describe sex differences in concussion characteristics of US Service Academy cadets using data collected from the CARE Consortium. The CARE Consortium represents the largest study of concussion to date, including outcomes data from 2210 concussions in cadets across the four US service academies: the Marines, the Naval Academy, the Air Force Academy, and the Coast Guard Academy.

## Methods

### Design and Setting

Data utilized in the current study were obtained for secondary analysis from the Concussion Assessment, Research, and Education (CARE) Consortium. The structure and procedures of the CARE Consortium have been described in detail elsewhere [17]. In brief, the CARE Consortium quantified sports-related concussion incidence and provided a longitudinal natural history of SRC in a large sample of collegiate athletes. CARE is a multisite study of 30 collegiate institutions (26 civilian institutions and 4 military service academies) that have enrolled over 41,000 military cadets and student–athletes (about 90% of all eligible athletes) and registered over 2500 SRCs. Participants completed a preseason baseline evaluation consisting of demographics, medical history concussion-like symptoms, postural control, and neurocognitive functioning. Concussions were diagnosed by a local team physician/athletic trainer.

For this study, data were obtained from concussions acquired by participants at the four US service academy sites from 2015 to 2019. This study was approved by the institutional review board at each study site, in conjunction with approval from the US Army Human Research Protection Office. All participants provided written informed consent prior to data collection. All data were collected in accordance with the Helsinki Declaration.

### Participants

At baseline testing, all US Service Academy cadets were asked to self-report their sex as either female or male. Male and female US Service Academy cadets who sustained at least one concussion during the study period were included in our analyses. One participant did not self-report sex and was, therefore, excluded from analyses. The final sample included 2209 concussions from 1947 cadets. This included 867 (39%) concussions from 752 (39%) female cadets and 1342 (61%) concussions from 1194 (61%) male cadets.

### Concussion Characteristics

For all CARE Consortium participants who sustained a concussion, the clinician completed an injury description at the first postinjury assessment. This injury description included the injury date and time and whether: (1) the cadet was injured during practice/training, competition, or outside of sport (e.g., car accidents, falling, or participation in recreational activities), including the detailed injury situation; (2) the cadet exhibited an altered mental status (e.g., dazed, stunned, or confused), LOC, PTA, RGA, or motor impairment (e.g., step, stumble, or fall) at the time of injury; (3) the cadet immediately reported the injury and was immediately removed from play; and (4) there was a delayed onset of symptoms after the injury, among other sport-specific items (e.g., playing surface). In addition, we examined the cadets’ sport type (i.e., contact, limited-contact, noncontact NCAA athletes, or non-NCAA athlete (noncollegiate athletes), whether the concussion represented a recurrent injury in the dataset (i.e., was not the first concussion for that particular athlete), and if the cadet exhibited a slowed recovery trajectory (i.e., greater than 14 days to initiate the return to play protocol and/or greater than 24 days for unrestricted return to activity) [18, 19].

### Statistical Analysis

We calculated injury proportion ratio (IPR) to determine sex differences in concussion characteristics. The IPRs represent a comparison of the proportion of concussions with certain characteristics between two groups (i.e., male and female cadets). The IPRs were defined as:

$$\text{IPR}= \frac{\frac{\text{no}.\text{ of concussions from character of interest in male cadets}}{\text{no}.\text{ of total concussions in male cadets}}}{\frac{\text{no}.\text{ of concussions from character of interest in female cadets}}{\text{no}.\text{ of total concussions in female cadets}}}$$

We also calculated 95% confidence intervals (CIs) for the IPRs, whereby those IPRs with 95% CIs not containing 1 were considered significant.

## Results

### Sex Differences in Concussions by Injury Situation

**Fig. 1:**
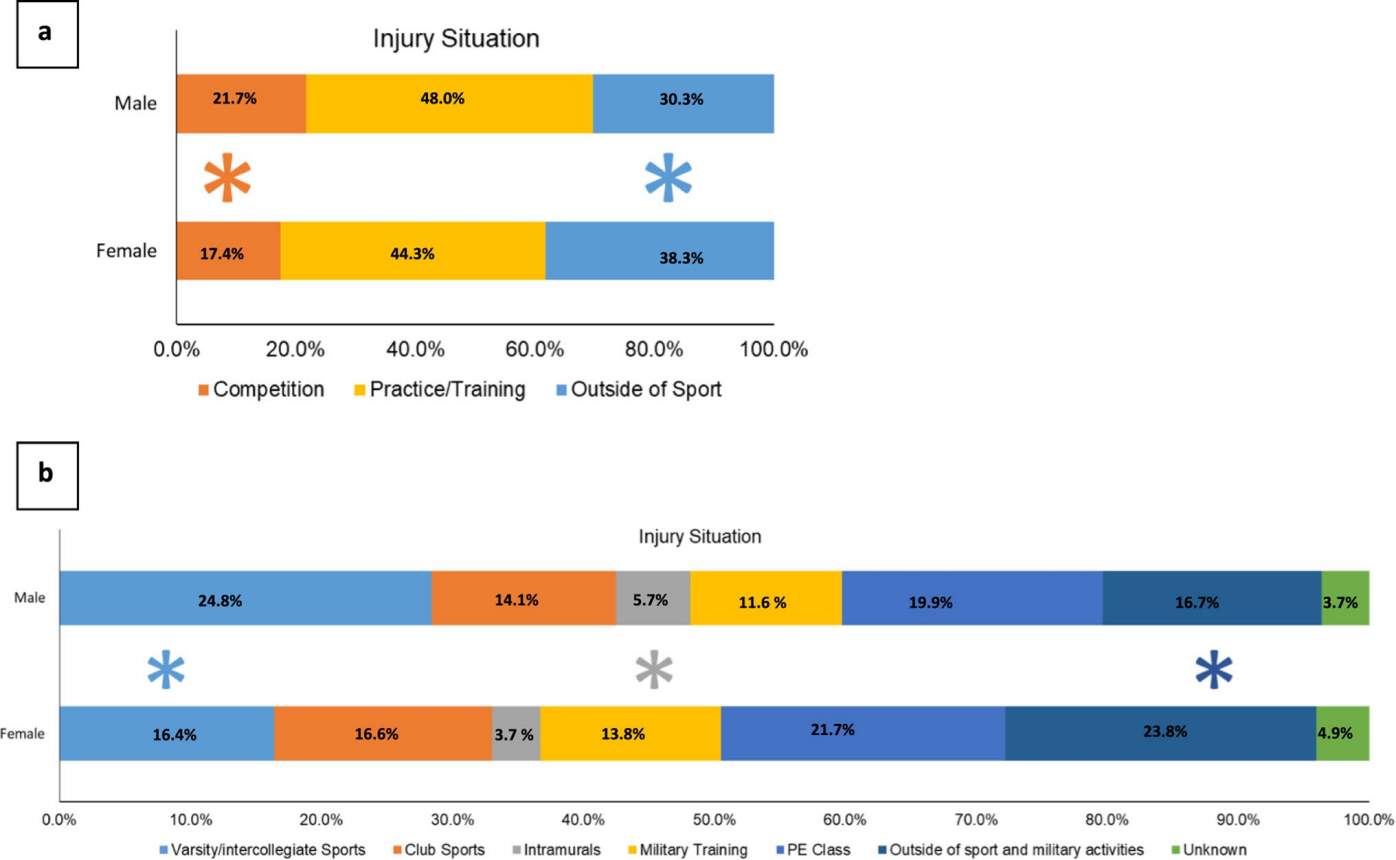
Sex differences in concussion characteristics by injury situation. ** a** Concussions from competition accounted for a larger proportion of concussions in males than in females, whereas concussions from outside of sport accounted for a smaller proportion of concussions in males than in females. ** b** Concussions from varsity/intercollegiate sports and intramurals accounted for a larger proportion of concussions in males than in females, whereas concussions from activities outside of sport and military training accounted for a larger proportion of concussions in females than in males. *Indicates statistically significant differences between sexes

Concussions from competition [IPR of 1.25 (95% CI 1.02–1.52)] accounted for a larger proportion of concussions in males than in females, whereas concussions from outside of sport [IPR of 0.79 (95% CI of 0.69–0.92)] accounted for a smaller proportion of concussions in males than in females (Fig. 1a). More specifically, concussions from varsity/intercollegiate sports [IPR of 1.73 (95% CI of 1.43–2.10)] and intramurals [IPR of 1.53 (95% CI 1.02–2.32)] accounted for a larger proportion of concussions in males than in females, whereas concussions from activities outside of sport and military training [IPR of 0.70 (95% CI 0.58–0.85)]​ accounted for a larger proportion of concussions in females than in males (Fig. 1b).

### Concussions with Altered Mental Status and Reporting Time

**Fig. 2:**
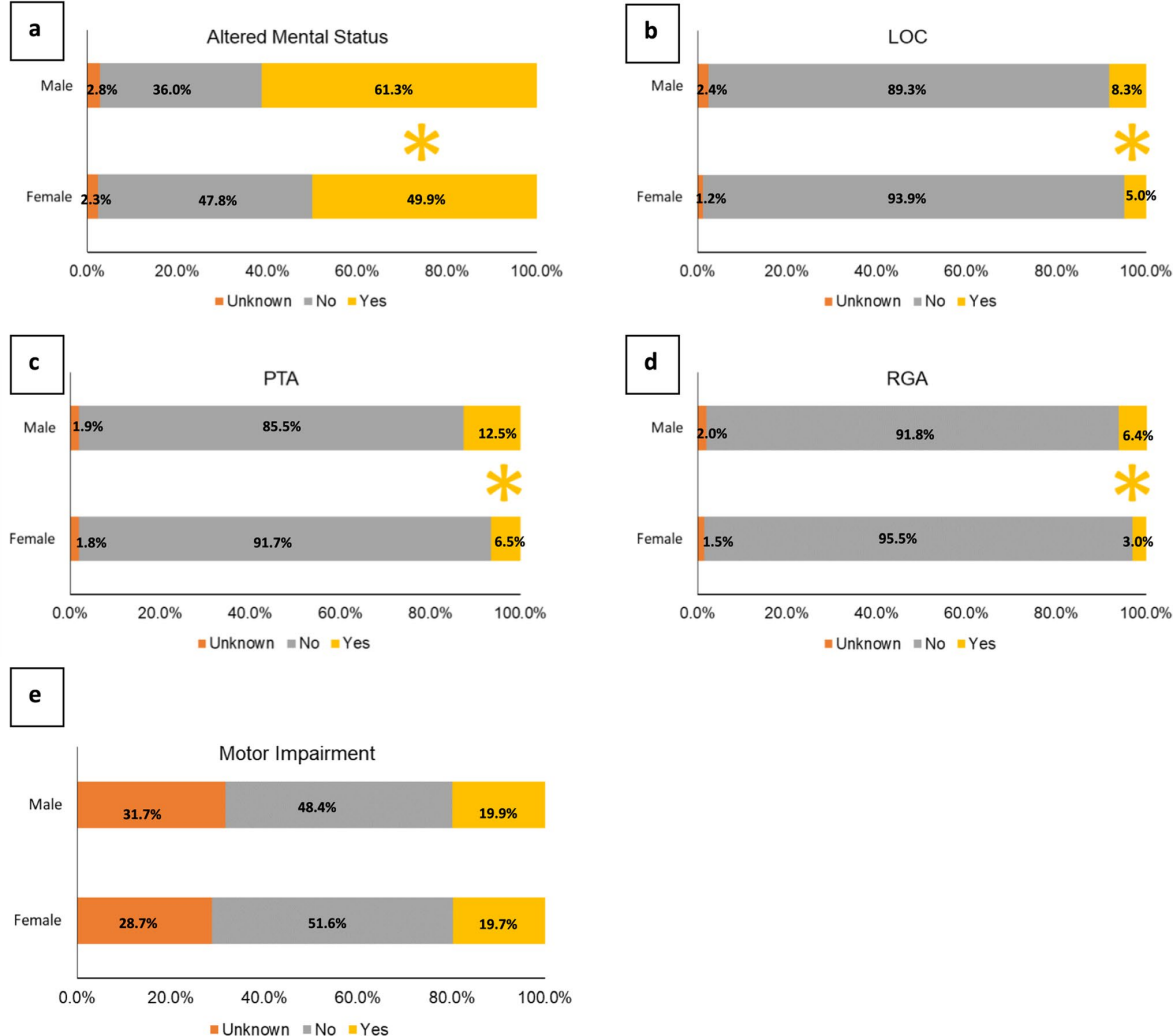
Sex differences in concussion characteristics by observable clinical signs. Concussions with altered mental status (**a**), loss of consciousness (LOC) (**b**), posttraumatic amnesia (PTA) (**c**), and retrograde amnesia (RGA) (**d**) accounted for a larger proportion of concussions in males than in females. **e** There was no difference in the proportion of concussions with motor impairments between male and female cadets. *Indicates statistically significant differences between sexes

**Fig. 3:**
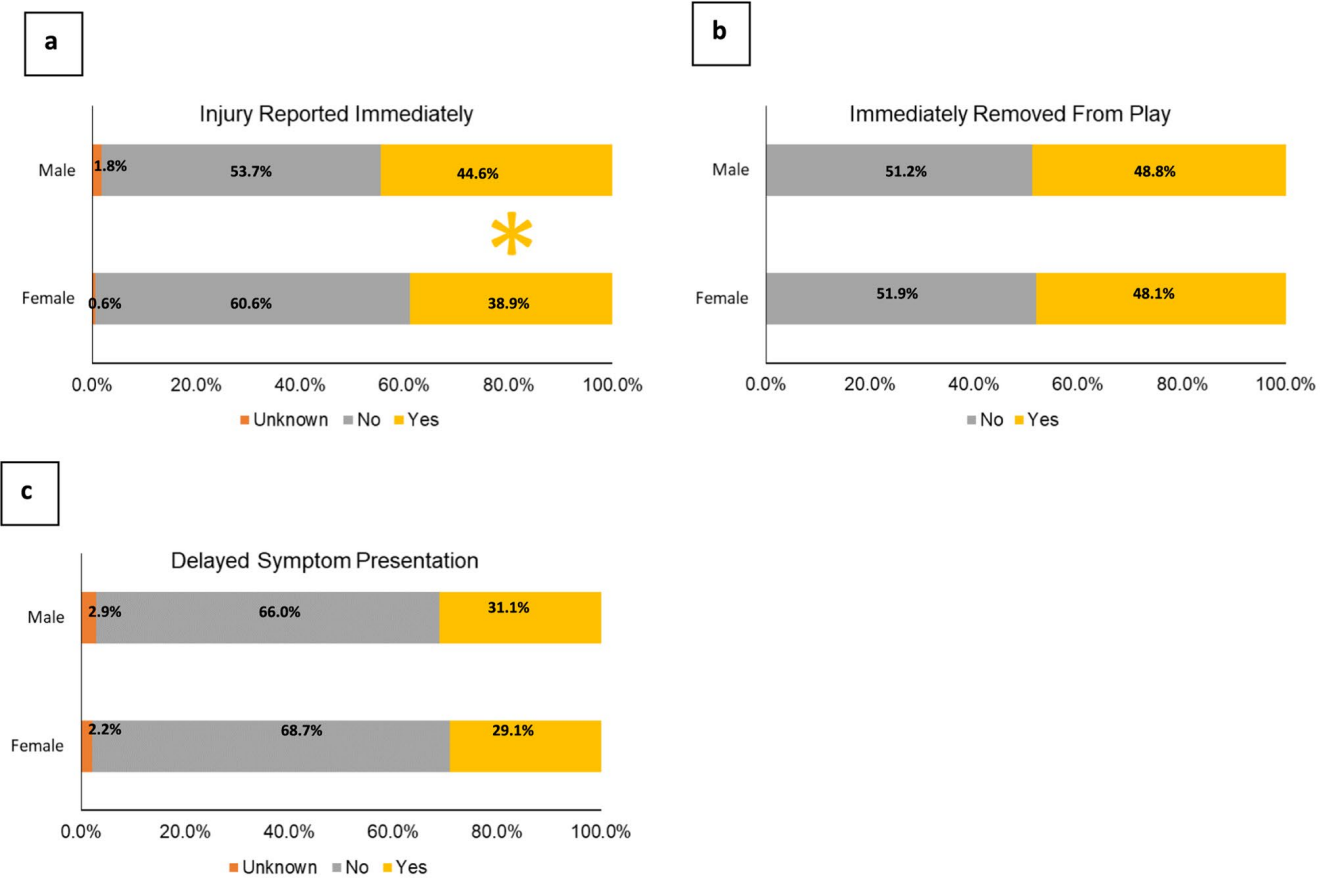
Sex differences in concussion characteristics by reporting time (**a**), time to removal from play (**b**), and delayed symptom presentation (**c**). Concussions reported immediately accounted for a larger proportion of concussions in males than in females, but there was no difference in the proportion of concussions immediately removed from play or with delayed symptom onset between male and female cadets. *Indicates statistically significant differences between sexes

In addition, a larger proportion of concussions in males resulted in altered mental status [IPR of 1.23 (95% CI 1.09–1.38)], LOC [IPR of 1.67 (95% CI 1.17–2.37)], PTA [IPR of 1.94 (95% CI 1.43–2.62)], and RGA [IPR of 2.14 (95% CI 1.38–3.31)] in contrast to females (Fig. 2). There was no difference in the proportion of concussions with motor impairments between male and female cadets. Further, a greater proportion of males reported their concussion immediately [IPR of 1.15 (95% CI 1.00–2.31)] than females; however, there was no difference in the proportion of concussions immediately removed from play or with delayed symptom onset between male and female cadets (Fig. 3).

### Concussions by Sport Type, Recurrent Concussions and Slow to Recover

**Fig. 4:**
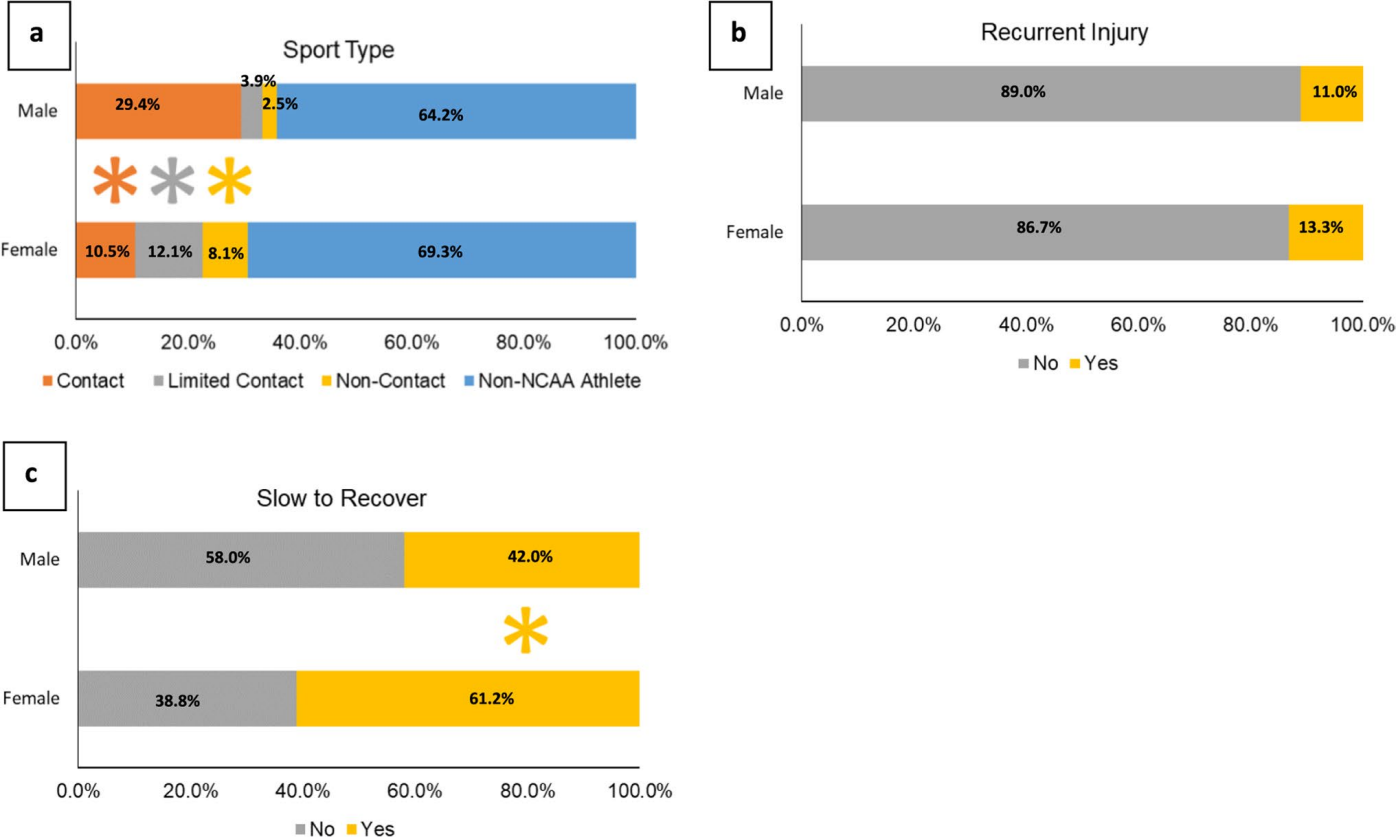
Sex differences in concussion characteristics by sport type (**a**), recurrent injury (**b**), and slow to recover (**c**). Concussions from contact sports accounted for a larger proportion of concussions in males than in females, whereas concussions from limited-contact and non-contact sports accounted for a smaller proportion of concussions in males than in females. There was no difference in the proportion of recurrent concussions between male and female cadets, although concussions that were slow to recover accounted for a smaller proportion of concussions in males than in females. *Indicates statistically significant differences between sexes

Concussions occurring from contact sports [IPR of 2.23 (95% CI 2.23–3.52)] accounted for a larger proportion of concussions among males than females, whereas concussions occurring from limited-contact [IPR of 0.23 (95% CI 0.23–0.45)] and noncontact sports [IPR of 0.20 (95% CI 0.20–0.46)] accounted for a smaller proportion of concussions among males than females (Fig. 4a). There was no difference in the proportion of recurrent concussions between male and female cadets (Fig. 4b); however, concussions resulting in prolonged recovery accounted for a smaller proportion of concussions in males than in females [IPR of 0.69 (95% CI 0.60–0.78), Fig. 4c].

## Discussion

A greater understanding of sex differences in concussion characteristics is needed to inform on-going risk, prevention, and management strategies given the increasing representation of women in the US military, including military training environments and sports participation, that inherently increases their concussion risk. Thus, the goal of this study was to describe sex differences in concussion characteristics of US Service Academy cadets using data collected from the CARE Consortium. As expected, notable differences across sexes regarding how concussions occurred were found. Specifically, female cadets represented a significantly greater proportion of those who did not report their concussion immediately and those who had a protracted recovery period. This is of significant concern as previous research in military cadets and intercollegiate athletes has shown delayed symptom reporting results in high acute symptom burden and increased time to asymptomatic recovery [10, 20–22]. Understanding why female cadets may delay reporting and factors that contribute to their longer recovery has significant implications for concussion safety protocols and educational efforts.

In this sample, female cadets also represented a significantly larger proportion of concussions from activities outside of sport and military training, as well as concussions with longer recovery times. While prior work from our group has shown non-sport related concussions (non-SRC) are associated with delayed symptom onset and longer recovery times in collegiate athletes, we did not see any differences in the proportion of male and female cadets presenting with delayed symptom onset [23]. It is possible cadets who sustain concussions outside of competitive sport or military training may be less likely to have access to on-site medical personnel trained in identifying concussion, delaying recognition, and reporting of the injury, which in turn leads to longer recovery times. Indeed, prior work suggests that when there is consistent access to on-site medical care, there are no reported differences in recovery patterns between female and male athletes [5]. Therefore, we believe that while delayed reporting alone is associated with prolonged recovery, it is possible that female cadets were more likely to display a longer recovery period due to a nonsports related injury mechanism. These findings are consistent with prior investigations among the general adult population that have shown that non-SRC is associated with persisting postconcussion symptoms, potentially due to injury biomechanics associated with nonsport related injuries [24].

Male cadets were more likely to display observable signs after their injury such as altered mental status, LOC, RGA, and PTA, compared with female cadets. This may relate to our findings described above, with these signs possibly having been easier for clinicians to identify relative to self-reported injuries. This, combined with the greater proportion of concussions in males occurring during competition, likely led to immediate removal and medical evaluation following the injury. While male cadets displayed more observable signs of a concussion, we found no difference between male and female cadets in the proportion of those immediately removed from play after a sports-related concussion, suggesting there is an alternative explanation for the reporting and recovery differences. For instance, Caccese and colleagues found nearly 28% of female cadets, compared to only 17% of male cadets, reported baseline symptoms that met the ICD-10 criteria for postconcussion syndrome (PCS) in the absence of an injury [25]. The greater endorsement of PCS symptoms at baseline, coupled with the lack of observable signs and resultant delayed reporting and longer recovery times, may make diagnosing concussion more difficult in female cadets. Future educational efforts should focus on clarifying additional signs and symptoms of concussions outside of observable signs as a potential method for increasing immediate report of an injury, especially considering that greater concussion knowledge is associated with greater intention of injury disclosure [26–29].

The findings in this sample are like those reported by Bookbinder and colleagues [16], where a smaller sample of cadets enrolled in the CARE Consortium study were analyzed and it was concluded that female cadets were nearly twice as likely to delay reporting in contrast to their male counterparts. However, other studies assessing likelihood of reporting an injury either revealed no sex-based differences [26, 30] or that females were more likely to report a concussion than their male counterparts [3, 31–33]. Thus, it is possible that the findings in this sample are unique to the environment as US service academies are exclusive spaces and the culture and environment of such academies and facilities are unfamiliar to most civilians. Furthermore, the findings may be unique due to the study design, as we were only able to examine immediate versus delayed reporting. It is possible that there was an intentional lack of reporting of a concussion by male cadets enrolled in the study, resulting in unreported concussions, and representing an important limitation in interpreting these findings. There is also a strong possibility that female cadets also intentionally underreported concussions. This underreporting may be a direct result of a personal perception of negative outcomes due to the culture of the US military. As females are the minority sex in US service academies, they may feel that reporting a concussion could jeopardize their academic grades and their reputation would be negatively affected. Future work should not only assess changes in cadet attitudes toward reporting longitudinally but also query reasons for delayed reporting after an injury to identify modifiable cultural or environmental factors.

### Clinical Implications

Understanding concussion reporting behaviors is important, as more than 30% of concussions go unreported [34, 35], which can dramatically affect medical support, care, and athlete recovery [35, 36]. The delayed reporting found in female cadets in this sample may represent a mechanism underlying the slower recovery in female athletes. However, a variety of factors explored in this sample, such as mechanism or setting of injury, signs and symptoms present after injury, and cultural and environmental aspects unique to the US military, represent targets for future educational efforts and concussion protocols to diminish sex differences in reporting and recovery patterns. Ultimately, these findings highlight injury characteristics that must be considered when accounting for any sex-based differences in concussion. Future work should examine different educational strategies targeting these modifiable factors to increase reporting of concussion and identification of symptoms after injury regardless of sex to potentially reduce the potential for prolonged recovery and negative long-term outcomes among military personnel.

### Limitations

As mentioned above, it is possible that information about all participants with concussion may not have been collected due to underreporting or delayed reporting that may have caused some injuries to be missed. It is worth noting that additional biopsychosocial factors may influence these relationships (e.g., race, ethnicity, socioeconomic status, and psychological state). In addition, this study focuses on cadets in military academies and thus, the results cannot be generalized to cadets outside of these academies or among collegiate athletes. Finally, we were unable to differentiate sex and sex, and future work should examine the intersection of these two variables in conjunction with extrinsic factors to develop better interventions for reducing sociocultural disparities in concussion characteristics. This topic provides a unique challenge, but also an opportunity for researchers to strive for better understanding of the differential influence of sex, especially in varied settings like sport and military service where a multitude of factors influence reporting behaviors and care-seeking behavior in general.

## Conclusions

A higher proportion of concussions occur outside of sport and military training for female cadets, who also displayed proportionally longer recovery times than males, despite males having higher severity markers (i.e., LOC, PTA, and RGA). Possible factors may include different mechanisms of injury outside of sport and military training, different biopsychosocial states associated with sex or injury context, and delayed injury reporting when outside of an observed environment.

## Supplementary Information

Below is the link to the electronic supplementary material.

Supplementary file1 (XLSX 17 KB)

## Declarations

### Availability of Data and Materials

Deidentified CARE data can be accessed via the Federal Interagency Traumatic Brain Injury Research database https://fitbir.nih.gov.

### Code Availability

N/A.
